# Study of DQE dependence with beam quality on GE Essential mammography flat panel

**DOI:** 10.1120/jacmp.v12i1.3176

**Published:** 2010-11-25

**Authors:** Rafael García‐Mollá, Rafael Linares Rafael Ayala

**Affiliations:** ^1^ Servicio de Dosimetría y Radioprotección Hospital General Universitario Gregorio Marañón Madrid Spain

**Keywords:** DQE, digital mammography, flat panel, beam hardening, IEC‐62220‐1‐2

## Abstract

This paper deals with the analysis of the behavior of objective image quality parameters for the new GE Senographe Essential FFDM system, in particular its dependence with beam quality. The detector consists of an indirect conversion a‐Si flat panel coupled to a CsI:Tl scintillator. The system under study has gone through a series of relevant modifications in flat panel with respect to the previous model (GE Senographe DS 2000). These changes in the detector modify its performance and are intended to favor advanced applications like tomosynthesis, which uses harder beam spectra and lower doses per exposure than conventional FFDM. Although our system does not have tomosynthesis implemented, we noticed that most clinical explorations were performed by automatically selecting a harder spectrum than that of typical use in FFDM (Rh/Rh 28–30 kV instead of Mo/Mo 28 kV). Since flat‐panel optimization for tomosynthesis influences the usual FFDM clinical performance, the new detector behavior needed to be investigated. Therefore, the aim of our study is evaluating the dependence of the detector performance for different beam spectra and exposure levels. In this way, we covered the clinical beam quality range (Rh/Rh 28–30 kV) and we extended the study to even harder spectra (Rh/Rh 34 kV). Detector performance is quantified by means of modulation transfer function (MTF), normalized noise power spectrum (NNPS) and detective quantum efficiency (DQE). We found that flat‐panel optimization results in slightly – but statistically significant – higher DQE values as beam quality increases, which is contrary to the expected behavior. This positive correlation between beam quality and DQE is also diametrically opposite to that of the previous model by the same manufacturer. As a direct consequence, usual FFDM takes advantage of the changes in the detector, as less exposure is needed to achieve the same DQE if harder beams are used.

PACS number: 87.59.ej

## I. INTRODUCTION

In order to quantitatively assess the image quality of digital systems, a set of parameters is employed, namely, signal transfer property (STP), modulation transfer function (MTF), normalized noise power spectrum (NNPS) and detective quantum efficiency (DQE).

This study deals with the assessment of objective image quality metrics for the new Senographe Essential full field digital mammography (FFDM) system, manufactured by GE Healthcare (Milwaukee, WI) which has been optimized with respect to a previous model (GE Senographe DS 2000). Both of them are indirect type flat‐panel detectors (CsI) and they both have the same del size (100 μm) but different detector size: Senographe Essential, $24\times 30.7\;{\text{cm}}^{2}$ and Senographe DS, $23\times 19.2\;{\text{cm}}^{2}$. As well, some improvements have been implemented in the latest GE mammographic detector that enhance the new detector for its potential use in tomosynthesis.^(^ ^1^ ^–^ ^3^ ^)^ To determine how clinical FFDM is influenced by these modifications, a survey of mammographic screening carried out by the system was performed. Data from mammographic technique factors were collected and some conclusions were drawn. The most common beam quality was a Rh/Rh (anode/filter) combination at 28–30 kV, a higher energy spectrum than the commonly used in typical mammograms (Mo/Mo 28 kV). This led us to the calculation of DQE at 28 kV, Rh/Rh, and then to the detailed study of the rest of parameters, and their dependence with beam‐quality and air kerma at detector entrance (KAD).

In digital mammography, several authors have compared different technologies by means of these image quality parameters and have studied how they are related to the visualization of contrast‐detail curves, as well.^(^ ^4^ ^–^ ^7^ ^)^ GE Senographe Essential image quality was previously assessed by C. Ghetti et al.^(^ ^8^ ^)^ at standard beam quality, Mo/Mo 28 kV, and then compared to the previous GE detector. There are few studies on the response of these parameters with the variation of the beam quality, among which it is worth pointing out the works by Suryanarayanan^(^ ^9^ ^)^ performed on a prototype FFDM sensor and Marshall,^(^ ^10^ ^)^ where DQE response with automatic exposure control is studied for two systems: the previous GE detector (Senographe 2000D) and a selenium device.

In this paper we aim to evaluate the new detector performance for different beam spectra and exposure levels, and therefore analyze the consequences of flat‐panel optimization in 2D mammography. In order to evaluate the equipment, a software tool (MIQuaELa v.1.0, Medical Image Quality Evaluation Laboratory)^(^ ^11^ ^,^ ^12^ ^)^ has been implemented in a high‐level language (MATLAB R2007b, The MathWorks, Inc.) that allows a routine assessment of NNPS, MTF and DQE, in compliance with International Electrotechnical Commission (IEC) 62220‐1‐2:2007 standard.^(^ ^13^ ^)^

## II. MATERIALS AND METHODS

### A. Equipment

The studied system was installed and passed the acceptance tests in November 2007. From that time on, annual and general QC tests have been performed according to the Spanish national digital mammography SEFM protocol based on 2006 European Guides, ACRIN and NHSBSP.^(^ ^14^ ^–^ ^16^ ^)^

The kilovoltage applied to the X‐ray tube (GE Apollon model) was kept within ±1 kV of the nominal value across the 25 kV to 34 kV range. X‐ray output at 100 cm was 33.58 μGy/mAs for a nominal tube potential of 28 kV, Molybdenum anode and 30 μm of Molybdenum filtration. System half value layer (HVL), at the same beam quality and anode/filter combination, measured with the compression plate out of the beam, was 0.35 mm Al equivalent.

The GE Senographe Essential uses an indirect conversion flat panel in which X‐rays are first converted to light by a scintillation material (CsI:Tl), and then converted to charge by an amorphous silicon (a‐Si) photodiode layer. Readout of the charge of each detector element is achieved using an active matrix thin film transistor (TFT array). The $24\times 30.72\;{\text{cm}}^{2}$ detector is mapped onto a 2400×3072 TFT array, resulting in a 100 μm del size and a Nyquist frequency of $5\;{\text{mm}}^{-1}$ relative to the del pitch.

The detector includes a series of modifications with respect to the previous model (GE Senographe 2000): (1) A storage capacitor has been added to each del to increase the charge storage capacity, enabling an extension of the maximum signal level and a reduction of the noise, (2) the time constant, RC, has been reduced, and (3) the CsI deposition has been optimized.^(^ ^1^ ^,^ ^2^ ^)^ Consequently, the maximum achievable exposure level has been increased; the noise – mainly at low doses – and lag effects have been reduced. This set of improvements, which are of great utility in advanced applications like tomosynthesis, will influence FFDM performance.

### B. Measurements

Objective image quality parameters (MTF, NNPS and DQE), together with their uncertainties, were evaluated according to IEC standard, specific for mammography.^(^ ^13^ ^)^ Several beam qualities and anode/filter combinations were explored, namely Mo 28 kV filtered with 30 μm of Molybdenum and Rh/Rh 26, 28, 30 and 34 kV filtered with 25 μm of Rhodium. With this selection of beam qualities we aimed to study the detector response under conditions of increasing beam energy by changing anode/filter combinations and/or applied tube voltage.

The KAD measurements were evaluated by using a calibrated Radcal 9015 dosimeter with its Radcal 10X5‐6M mammographic ionization chamber (Radcal Corporation, Monrovia, CA). All measurements were made according to the IEC standard geometry^(^ ^12^ ^)^ with the compression plate out of the beam, the grid removed and 2 mm Al filtration, 99.9% pure, added to the beam.

All the acquired images were analyzed in DICOM format as RAW data. Software image quality enhancement was disabled by switching off the Fine View and Premium View options in the acquisition console. Fine View restores the loss in spatial resolution caused by the blurring introduced by the phosphor by performing a SNR‐dependent MTF optimization (technical specifications of GE Senographe Essential). Premium View utility enhances local contrast of breast structures.^(^ ^7^ ^)^

### C. Signal transfer property (STP)

The signal transfer property, or detector response, states the relationship between mean pixel value (MPV) and KAD. Several uniform images were acquired at different exposure levels with a collimated X‐ray beam of $150\times 150\;{\text{mm}}^{2}$ at detector entrance. MPV was evaluated in a $50\times 50\;{\text{mm}}^{2}$ region of interest (ROI) placed at a distance of 50 mm from the chest wall edge where the radiation field is as uniform as possible. Kerma measurements were made in the same region used for evaluating MPV with a stainless steel plate placed at detector entrance, avoiding unnecessary radiation damage to the detector due to repeated exposures. Kerma at detector entrance was evaluated from these data correcting the chamber reading for the inverse square law. Values of selected nominal mAs range from 4 mAs up to 100 mAs, at all beam qualities. The detector response (MPV vs. KAD) was fitted to a linear equation^(^ ^17^ ^)^ whereby the degree of linearity is indicated by the correlation coefficient.

### D. Modulation transfer function (MTF)

The presampling MTF was measured using the edge technique, following the procedures described in IEC standard.^(^ ^13^ ^)^

The test device consisted of a 1 mm thick copper plate, enough for assuring radiopacity. The sharpness of the edge device was evaluated by means of a conventional screen film mammography (AD‐M Fuji). The plate was put in contact with the detector cover with edges angled at 2°–3° with respect to lines/columns of pixels. A region of interest (ROI) of $25\times 50\;{\text{mm}}^{2}$ was utilized for analysis.

In order to remove the effect of the nonuniformity of the radiation field, the original image pixel values were divided by a fitted 2D second order polynomial to the image data, following IEC recommendations. Previous linearization of data was not necessary, as the detector response is already linear.

In order to determine the presampling MTF, the edge image is divided into several groups of lines across the edge. The number of lines per group (N) and consequently the value of the angle, N=1/tan (α), need to be known. The angle is determined by a three‐step procedure: (1) edge determination using the Sobel operator which computes an approximation of the gradient of the image intensity function, (2) application of linear least squares method for data fitting, and (3) calculation of the line slope which equals tan (α). The subsequent vertical sampling of each of the N groups of lines leads to N representations of the oversampled edge spread function (ESF) that are later averaged. This helps to reduce the systematic and stochastic error. Due to the tilt angle, the edge position in the various edge profiles is different. The first pixel in the first line of each group is shifted a distance equal to pixel size, in line direction, with respect to the preceding pixel. Therefore, it is necessary to make a correction in pixels‐to‐edge distances before averaging. We have to bear in mind that the number of lines N is not an integer number, which could result in a cumulative offset affecting the average.^(^ ^18^ ^)^

The oversampled line spread function (LSF) was derived from the oversampled ESF by finite element differentiation using a [‐1, 0, 1] kernel. To obtain the MTF, a fast Fourier transform (FFT) was applied to the oversampled LSF. The magnitude of the FFT was normalized to 1 for zero frequency, and corrected for the transfer function of the finite‐element differentiation.^(^ ^19^ ^)^

In order to reduce statistical uncertainty, measurements were repeated up to 16 times, and MTF mean value and standard deviation were computed.

### E. Normalized noise power spectrum (NNPS)

The NPS was calculated according to IEC 62220‐1‐2 which establishes an area of analysis containing a minimum of 4 million independent pixels.^(^ ^13^ ^)^

For each ROI, slowly varying spatial background effects including the heel effect were corrected by fitting and subtracting a two‐dimensional second‐order polynomial to the original acquired image data. The area of analysis was subsequently divided into sub‐ROIs of 256×256 pixels, overlapped by 128 pixels in both horizontal and vertical directions. The 2D noise power spectrum (NPS) was calculated by using the equation below:^(^ ^13^ ^)^

(1) $$\mathrm{NPS}(u_{n},v_{k})=\frac{\mathrm{\Delta x\Delta y}}{M\times 256\times 256}\sum_{m=1}^{M}|\sum_{i=1}^{256}\sum_{j=1}^{256}\left(I\left(x_{i},y_{j}\right)-S\left(x_{i},y_{j}\right)\right)\exp\left(-2\pi \cdot i\left(u_{n}x_{i}+v_{k}y_{j}\right)\right){|}^{2}$$

where *u* and *v* are the spatial frequency variables, Δ*x* and Δ*y* are the pixel spacing in respectively the horizontal and vertical direction, *M* is the number of ROIs, $I(x_{i},y_{j})$ is the linearized data (mean pixel values transformed to KAD by means of the Signal Transfer Property), and $S(x_{i},y_{j})$ is the fitted two‐dimensional polynomial. NNPS is obtained by dividing NPS by the square of the corresponding KAD.

According to IEC standard,^(^ ^13^ ^)^ 15 rows or columns of the two‐dimensional spectrum around each axis are used for averaging, omitting both axes themselves, in order to determine the one‐dimensional (1D)‐NNPS, in both horizontal and vertical directions.

Calculations were performed using 10 uniform images, following the method described by N.W. Marshall.^(^ ^6^ ^)^

### F. Detective quantum efficiency (DQE)

The following equation was employed to calculate the DQE of the system:^(^ ^13^ ^)^

(2) $$\mathrm{DQE}(u)=\frac{{\mathrm{MTF}}^{2}(u)}{{\mathrm{SNR}}_{\mathrm{in}}^{2}\times \mathrm{KAD}\times \mathrm{NNPS}(u)}$$

where *KAD* is the measured air kerma at detector entrance and $SNR_{in}^{2}$ is the squared signal‐to‐noise ratio per air kerma. DQE uncertainty at the studied beam qualities has been calculated following the instructions contained in Guide to the Expression of Uncertainty in Measurement (GUM);^(^ ^20^ ^)^ measurements and error bars are expressed using a coverage factor value k=2. NNPS, KAD, ${\text{MTF}}^{2}$ and STP fit uncertainties were taken into account.

A subroutine from MIQuaELa v.1.0 package,^(^ ^11^ ^,^ ^12^ ^)^ was used to calculate the values of $SNR_{in}^{2}$ and HVL at any beam quality, with several selectable anode/filter combinations, voltages and added filtrations. $SNR_{in}^{2}$ is assessed just as stated in IEC standard Annex B.^(^ ^13^ ^)^ The method is based in the estimation of quantum fluence from the algorithm developed by Boone et al.,^(^ ^21^ ^,^ ^22^ ^)^ which yields the number of X‐ray photons per energy bin. For each of the energy bins, the filtration of the X‐ray beam is applied using attenuation coefficients from NIST.^(^ ^23^ ^)^ From this data and the calculated X‐ray quanta per unit exposure $SNR_{in}^{2}$ can be evaluated. In order to estimate the agreement between actual and calculated spectra, measured and simulated HVL were compared.

## III. RESULTS & DISCUSSION

### A. Signal transfer property (STP)

**Figure 1:**
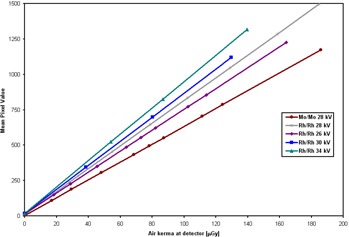
Signal transfer properties at the studied beam qualities.

The signal transfer properties of each anode/filter combination at 28 kV and for the same anode/filter combination (Rh/Rh) for different voltages are plotted in Fig. 1. Table 1 shows the linear fit parameters (slope, ordinate at origin and standard deviation (σ)), as well as HVL values for the studied beam qualities. As expected, the relationship between MPV and KAD is linear, with correlation coefficients larger than 0.999, in all cases. It can be seen that there is a direct relationship between the magnitude of the slope and HVL, with the highest slope corresponding to the hardest spectrum. In other words, an increase in spectrum hardness results in less KAD needed to produce the same pixel value. This behavior can be explained by the increase in cover transmission and photon fluence (at the same KAD) that takes place in the new detector for increasing beam qualities within the mammographic kV range. Data are in excellent agreement with those published by NHSBSP.^(^ ^17^ ^)^

**Table 1:** Slope and offset (and associated standard deviations) for the signal transfer property at several beam qualities (2 mm Al added filtration in all cases).

*Anode/filter*	*kV*	*HVL (mm Al)*	*Slope (1/μGy)*	*Offset*	*σ (Slope) (1/μGy)*	*σ (Offset)*
Mo/Mo	28	0.61	6.29665	2.92	0.00025	0.24
Rh/Rh	28	0.72	8.02481	12.96	0.00058	0.35
Rh/Rh	26	0.70	7.36799	15.81	0.00035	0.20
Rh/Rh	30	0.77	8.49660	17.72	0.00021	0.95
Rh/Rh	34	0.82	9.30451	18.76	0.00020	0.91

### B. Modulation transfer function (MTF)

**Figure 2:**
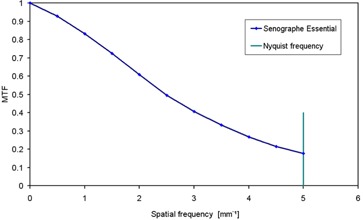
Presampling MTF of Senographe Essential as a function of spatial frequency.

Figure 2 shows the system presampling MTF that is essentially the same at all exposure levels and beam qualities. Vertical and horizontal MTFs were calculated and averaged following IEC standard for further calculations.^(^ ^12^ ^)^ Presampling MTF values at 2 lp/mm and 4 lp/mm are 0.600 and 0.268, respectively. These figures are in very good agreement with those obtained in the internal IQST test (0.605 and 0.276) and by other authors.^(^ ^8^ ^)^

MTF values are smaller than those found in the previous GE Senographe 2000 DS model (0.18 vs. 0.37 at Nyquist frequency).^(^ ^4^ ^,^ ^5^ ^)^ This decrease in MTF with respect to the previous GE model may be understood according to the changes introduced in the new system. Specifically, the CsI:Tl scintillator thickness is increased in order to enhance DQE at low spatial frequencies. This results in more light scattering in the needle‐structured phosphor and, hence, a reduction in MTF.^(^ ^24^ ^)^

### C. NNPS

**Figure 3:**
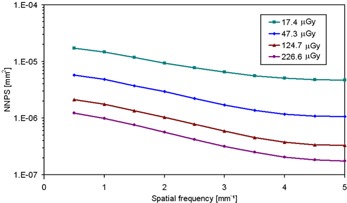
NNPS at four different KAD values for 28 kV, Mo/Mo anode/filter combination.

Figure 3 shows 1D NNPS at several values of air kerma for Mo/Mo combination at 28 kV. It is clear that NNPS values exhibit a dependence on exposure, and decrease with increasing air kerma. Moreover, the values are small as compared to other flat panels, mainly at low KAD values within the tomosynthesis range.^(^ ^4^ ^–^ ^6^ ^)^

**Figure 4:**
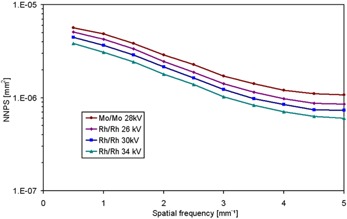
Comparison of experimentally measured NNPS for MoMo 28 kV and Rh/Rh 26, 30 and 34 kV at approximately 50 μGy KAD.

In Figure 4, 1D NNPS curves versus spatial frequency are plotted for Mo/Mo at 28 kV, Rh/Rh at 26, 30 and 34 kV anode/filter combinations, at approximately the same KAD value, 50 μGy; in particular: 47.98, 51.01, 49.32 and 53.70 μGy, respectively. As in the rest of comparisons, KAD values are not exactly the same at all beam qualities, since mAs are only selectable on a discrete basis. It can be seen that NNPS is lower for Rh/Rh combination than for Mo/Mo. We also see that, maintaining the same anode/filter combination (Rh/Rh), NNPS values are lower for higher voltages at all spatial frequencies. This decrease in NNPS with increasing beam hardness can be explained by: (1) the higher photon fluence when using a harder beam (Rh/Rh instead of Mo/Mo for the same kV, and 34 kV instead of 26 kV for the same anode/filter combination) at the same KAD, which implies a smaller influence of electronic noise, and (2) the higher mean energy of the spectrum that leads to a higher transmission of the flat‐panel cover.

### D. DQE

Table 2 shows calculated values of $SNR_{in}^{2}$ and HVL at the studied beam qualities. Spectral differences between calculations and measurements are estimated by means of the first HVL. Actual and simulated HVL values agree within 2.8% in the worst case.

**Table 2:** Measured and calculated first HVL (mm Al) at the studied beam qualities, and difference between both. Calculated values of $SNR_{in}^{2}$ are also shown.

*Anode/filter*	*kV*	*HVL Measured (mm Al)*	*HVL Software Tool (mm Al)*	*HVL difference (%)*	$SNR_{in}^{2}$ *Software Tool* $\left(1/(mm^{2}\cdot \mu Gy)\right)$
Mo/Mo	28	0.61	0.60	1.6	5126
Rh/Rh	28	0.72	0.74	2.8	5847
Rh/Rh	26	0.70	0.70	0.0	5579
Rh/Rh	30	0.77	0.78	1.3	6081
Rh/Rh	34	0.82	0.83	1.2	6561

**Figure 5:**
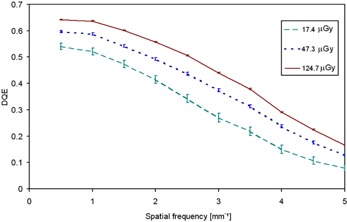
Experimentally measured DQE at three different KAD for 28 kV, Mo/Mo anode/filter combination.

DQE variation with air kerma at Mo/Mo 28 kV is shown in Fig. 5. DQE (maximum) values reach 0.647 at 124.7 μGy and 0.609 at 47.3 μGy but fall to 0.166 and 0.128, respectively, at Nyquist frequency. The rapid decrease of DQE with increasing spatial frequency in this flat panel is related to the shape of the MTF. DQE curves have been plotted according to IEC 62220‐1:2004^(^ ^25^ ^)^ with their corresponding error bars.

**Figure 6:**
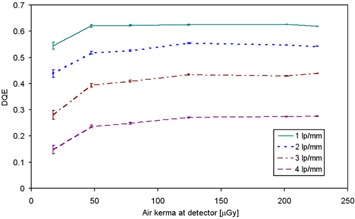
DQE as a function of KAD at four different spatial frequencies for 28 kV, Mo/Mo anode/filter combination.

Figure 6 shows DQE variation with kerma at four different spatial frequencies. It can be seen that DQE rapidly increases with KAD at low kerma values below 100 μGy, but it becomes insensitive to variations at higher doses; an increase of 1% is observed at kerma values higher than 130 μGy.

**Figure 7:**
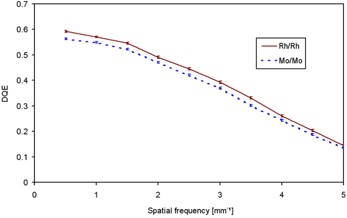
Experimentally measured DQE for 28 kV, Rh/Rh and Mo/Mo anode/filter combination, at approximately 77 μGy.

**Figure 8:**
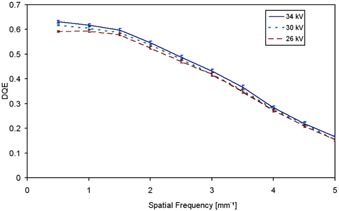
Experimentally measured DQE for Rh/Rh anode/filter combination, at three different voltages and approximately 50 μGy.

In Fig. 7, DQE values with their corresponding error bars at 28 kV for Mo/Mo and Rh/Rh combinations are plotted versus spatial frequency at KAD values of 77.42 and 77.11 μGy. At a first glance, the detector seems to be optimized for the hardest beam qualities, as DQE values for Rh/Rh are higher than that of Mo/Mo at all frequencies. In order to confirm this behavior, we extended the study to three values of kV, namely 26, 30 and 34 kV, for the same anode/filter combination (Rh/Rh) at 55.14, 50.04 and 54.50 μGy, respectively. Results are presented in Fig. 8. Differences are more evident at low frequencies. At higher frequencies, the DQE is less sensitive to changes in beam quality. Rather, it is strongly dependent on the MTF, which was shown to be independent of beam quality and KAD. In order to elucidate whether DQE increase is within the uncertainty of calculations and measurements, it was assessed by using 10 uniform images (for NPS assessment) and 16 MTF measurements. Thus, DQE uncertainty is reduced and a more statistically significant mean value is achieved. Geometry settings were kept unchanged, and the same averaged MTF was employed in all cases, so as to avoid other error sources.

Table 3 shows a summary of the relationship between measured HVL and DQE, together with the uncertainties involved. DQE variation with anode/filter combinations is clearer than that found by only changing kV values (keeping anode and filtration unchanged). Nevertheless, even in this case, error bars do not overlap in the low frequency zone for 26 and 34 kV, and we can conclude that there is a positive correlation between beam quality and DQE. This positive correlation between DQE and beam quality is diametrically opposite to that found in the literature for the previous flat panel manufactured by GE.^(^ ^26^ ^,^ ^27^ ^)^ This behavior is also contrary to the expected decrease in quantum detective efficiency (QDE) – due to the shape of the CsI:Tl absorption spectrum – when increasing beam quality at mammography kV levels.^(^ ^28^ ^)^

**Table 3:** DQE values and expanded uncertainty (cover factor K=2) for the studied anode/filter combinations and kV at 1 lp/mm and approximately 50 μGy (2 mm Al added filtration in all cases).

*Anode/Filter*	*kV*	*HVL Measured (mm Al)*	*DQE at 1 lp/mm Coverage Factor* k=2	$SNR_{in}^{2}$ $\left(1/(mm^{2}\cdot \mu Gy)\right)$
Mo/Mo	28	0.61	0.5854±0.0042	5126
Rh/Rh	28	0.72	0.5978±0.0057	5847
Rh/Rh	26	0.70	0.5937±0.0052	5579
Rh/Rh	30	0.77	0.6034±0.0042	6081
Rh/Rh	34	0.82	0.6142±0.0050	6561

Nevertheless, the different performance of the new detector model compared to the previous one can be explained by the Senographe Essential enhancement technique previously mentioned. First of all, the scintillator thickness has been increased, deposition techniques have been refined and the maximum achievable exposure level has been increased compared to the previous model. Increasing the scintillator thickness results in higher QDE values and consequently better DQE, mainly at low spatial frequencies.^(^ ^1^ ^)^ Secondly, and as stated in the NNPS section above, the harder the beam, the higher the photon fluence for the same KAD. Thirdly, a higher mean energy implies a higher cover transmission that, combined with the increase in fluence, is responsible for the higher signal reached in the detector. We can conclude that, due to the flat‐panel optimization, the increase in fluence and cover transmission compensates the decrease in absorption due to the shape of the CsI absorption spectrum. This compensation did not occur in the previous detector. The direct consequence is that, for same KAD, NNPS decreases with increasing beam energy, as much as to compensate for the higher $SNR_{in}^{2}$.

## IV. CONCLUSIONS

A comprehensive study of the dependence of detector image quality with exposure and beam quality has been carried out, and several conclusions can be drawn. The study of STP shows a clear correlation between slope and beam quality, which has a direct consequence in the detector performance, namely, the harder the beam within the spectral range tested, the less KAD is needed to achieve the same mean pixel value.

It has been shown that NNPS is low as compared to other detectors, mainly at low doses, and decreases with beam hardness. As a consequence, beam hardening within the studied range gives rise to higher DQE values, in contrast to what it is to be expected from the shape of the CsI:Tl absorption spectrum and the published data about the previous GE model.

The most significant results are the increase in DQE with increasing beam quality (0.618 for Rh/Rh 34 kV and 0.593 for Mo/Mo 28 kV at 50 μGy) and the fact that these values are close to the maximum DQE attained (0.647) with the Mo/Mo anode/filter combination, 28 kV, at 124.7 μGy.

The new features and improvements outlined above enhance the detector performance for its use in tomosynthesis.^(^ ^1^ ^,^ ^2^ ^)^ Moreover, clinical FFDM also takes advantage of detector characteristics – slight dose dependence and harder spectra optimization (Rh/Rh) – resulting in a high potential for reduction of dose delivered to patient.
